# Smoking and Colorectal Cancer in a Non-Western Population: a Prospective Cohort Study in Japan

**DOI:** 10.2188/jea.13.323

**Published:** 2007-11-30

**Authors:** Kenji Wakai, Norihiko Hayakawa, Masayo Kojima, Koji Tamakoshi, Yoshiyuki Watanabe, Koji Suzuki, Shuji Hashimoto, Shinkan Tokudome, Hideaki Toyoshima, Yoshinori Ito, Akiko Tamakoshi

**Affiliations:** 1 Division of Epidemiology and Prevention, Aichi Cancer Center Research Institute.; 2 Department of Preventive Medicine/Biostatistics and Medical Decision Making, Nagoya University Graduate School of Medicine.; 3 Department of Epidemiology, Research Institute for Radiation Biology and Medicine, Hiroshima University.; 4 Department of Health Promotion and Preventive Medicine, Nagoya City University Graduate School of Medical Sciences.; 5 Department of Public Health/Health Information Dynamics, Nagoya University Graduate School of Medicine.; 6 Department of Epidemiology for Community Health and Medicine, Kyoto Prefectural University of Medicine Graduate School of Medical Science.; 7 Department of Public Health, Fujita Health University School of Health Sciences.; 8 Department of Hygiene, Fujita Health University School of Medicine.

**Keywords:** smoking, colonic neoplasms, rectal neoplasms, cohort studies, Japan

## Abstract

BACKGROUND: The risk of colorectal cancer in relation to smoking habits has been examined mostly in Caucasians, and evidence for other ethnic groups is still scarce.

METHODS: Our data came from the Japan Collaborative Cohort (JACC) Study. From 1988 through 1990, 25,260 men and 34,619 women aged 40-79 years completed a questionnaire on cigarette smoking and other lifestyle factors. Hazard ratios (HR) were estimated by fitting proportional hazards models.

RESULTS: During the mean follow-up of 7.6 years through December 1997, we documented 408 incident colon cancers and 204 rectal cancers. We found a non-significant increase in colon cancer risk in male current smokers compared with never smokers. The multivariate-adjusted hazard ratios were 1.07 (95% confidence interval [CI]: 0.72-1.59) for ex-smokers and 1.23 (95% CI: 0.85-1.78) for current smokers. We however failed to observe a clear dose-response relationship between smoking intensity or duration and colon cancer risk. The adjusted hazard ratio was 1.07 (95% CI: 0.71-1.61) even for 40+ years of smoking. Almost no increase in colon cancer risk was detected for female smokers, and male smokers were not at an enhanced risk of rectal cancer.

CONCLUSIONS: Cigarette smoking was not a strong risk factor for colorectal cancer even after a long-term exposure, although a weak association remains open to discussion.

Carcinogens from tobacco smoke can reach the colorectal mucosa through the digestive tract^1^ or the circulatory system,^2^ and epidemiologic studies have consistently related colorectal adenoma, an established cancer precursor, with cigarette smoking.^3^ Although most of earlier studies did not find an increased risk of colorectal cancer in smokers, several recent investigations after 1990 have shown an association with tobacco use, raising the possibility that this cancer may be another tobacco-related malignancy.^3^ Taking into account the possible 35 to 40-year induction period,^4^ we should now be able to detect any increase in risk, because the main spread of the smoking habit occurred in the first half of the 20th century in developed countries.

The findings on this issue, however, remain quite inconsistent, and many studies have failed to find a clear association.^5^ Further, the risk of colorectal cancer in relation to smoking habits has been examined mostly in Caucasians, and evidence for other ethnic groups is still scarce.^3^ However, the incidence rates of colorectal cancer have generally been increasing in Asian countries,^6^ and identification of modifiable risk factors for the malignancy in this area is important.

We therefore examined the relationship between smoking and the risk of colorectal cancer in Japanese men and women, using data from the Japan Collaborative Cohort (JACC) Study, a nationwide prospective study.

## METHODS

The JACC Study started between 1988 and 1990, when 110,792 inhabitants aged 40 to 79 years completed a baseline questionnaire.^7^^,^^8^ They were enrolled from 45 study areas throughout Japan, mostly when they underwent municipal health checkups. Informed consent was obtained from them, and the Ethics Committee of Fujita Health University approved this investigation.

Subjects for the present analysis were restricted to 65,184 individuals who lived in 24 study areas, where cancer registries are available. Of the total, we excluded 58 with a previous history of colorectal cancer and 5,247 of unknown smoking status (never, former, or current smokers), leaving 59,879 eligible subjects (25,260 men and 34,619 women) for the analysis.

The baseline questionnaire covered lifestyle including smoking and drinking habits, physical activity, and consumption of selected foods, as well as medical history, education, family history of cancer, height and weight, and occupation held the longest. For cigarette smoking, participants were asked to describe their smoking status, age at starting smoking, the average number of cigarettes smoked per day, years of smoking, and years since smoking cessation (for ex-smokers). We did not examine smoking of tobacco other than cigarettes because cigar and pipe use in Japan is limited.^9^

We used population registries in the municipalities to determine the vital and residential status of the subjects. Registration of death is required by the Family Registration Law in Japan and is followed across the country. Because of logistical reasons, we discontinued the follow-up of subjects who moved out of the study areas.

We ascertained incidence of cancer by means of linkage with the records of population-based cancer registries, supplemented by systematic review of death certificates.^7^ The follow-up was conducted from the time of baseline survey through the end of 1997 except for three areas (to the end of 1994, 1995, and 1996, respectively). During the study period, only 2.7% (1,620) of the participants were lost to follow-up due to moving.

The mortality to incidence ratio for colorectal cancer was 0.28 in the cohort covered by cancer registries. In the present study, cases of colorectal cancer in mucosa were also registered: they accounted for 27.8% of all the cases with a known clinical stage. The mortality to incidence ratio would have been increased up to 0.35 if all the patients with cancer in mucosa had been alive and excluded. This figure, however, is still comparable with those in acceptably accurate population-based cancer registries in Japan (0.23 to 0.51)^10^ and indicates that reasonably high proportion of colorectal cancer cases were identified.

Body mass index at baseline survey was calculated based on the reported height and weight (body mass index = weight in kilograms/[height in meters]^2^). The cumulative amount of cigarette smoking was measured as pack-years, calculated by multiplying the number of packs smoked per day by years of smoking. We compared background characteristics between never, former, and current smokers by the one-way analysis of variance or the chi square test.

We counted person-time of follow-up for each participant from the date of filling out the baseline questionnaire to development of colorectal cancer, death from any cause, emigration outside the study area, or the end of follow-up period, whichever came first. Those who died from causes other than colorectal cancer or moved out of the study fields were treated as censored cases.

The hazard ratios (HRs) for colon or rectal cancer by sex and smoking status at baseline were estimated using proportional hazards models,^11^ with adjustment for age and other potential confounders,^12^ including area (Hokkaido and Tohoku, Kanto, Chubu, Kinki, Chugoku, or Kyushu), education (attended school until the age of <16, 16-18, or 19+), family history of colorectal cancer in parents or siblings (yes or no), body mass index (<20.0, 20.0-24.9, or 25.0+ kg/m^2^ for men, and <20.0, 20.0-24.9, 25.0-29.9, or 30.0+ kg/m^2^ for women), alcohol drinking (never drinkers, ex-drinkers, light current drinkers [<2.0 Japanese drinks or 44 g of ethanol per day], or heavy current drinkers [2.0+ Japanese drinks/day] for men, and never drinkers, ex-drinkers, or current drinkers for women), walking time (≤30 or 30+ minutes/day), sedentary work (yes or no), and consumption of green leafy vegetables (≤2 times/week, 3-4 times/week, or every day) and beef (almost never, 1-2 times/month, 1-2 times/week, or 3+ times/week). Missing values for each covariate were treated as an additional category in the variable and were included in the model.

The HRs according to detailed characteristics of smoking habits were also computed for male subjects. The cases of colorectal cancer in female ex- or current smokers were too few to estimate the ratios. We additionally attempted to consider total cigarette pack-years smoked by age of 30 years and those smoked after the age in relation to the risk of colorectal cancer.^4^ The risk was also associated with cumulative smoking until or after 20 years before baseline.^4^ A linear trend of association was assessed by the regression model assigning scores (0, 1, 2,…) to the levels of the independent variable. All p values were two-sided, and all the analyses were performed using the SAS^®^ (Cary, USA).^13^

## RESULTS

During the mean follow-up of 7.6 (standard deviation=1.9) years, we identified 408 cases of colon cancer (219 in men and 189 in women) and 204 cases of rectal cancer (147 in men and 57 in women).

Table 1 summarizes background characteristics of the subjects according to smoking status by sex. At baseline, proportions of never, former, and current smokers were 20.6%, 27.1%, and 52.3% in men and 93.1%, 1.6%, and 5.3% in women, respectively. Current smokers were likely to be lean and to consume less green leafy vegetables in both sexes, and tended to be less educated, particularly in men. Ex-smokers tended to be older and to report shorter walking time. Smoking habits were positively associated with current alcohol drinking in men and women.

**Table 1.** Background characteristics of subjects according to smoking status by sex.

Characteristics	Smoking status							

	Men				Women			
	
	Never	Ex-smokers	Current smokers	p	Never	Ex-smokers	Current smokers	p
	(n = 5,199)	(n = 6,851)	(n = 13,210)		(n = 32,226)	(n =570)	(n = 1,823)	
Age (years)^a^	57.4 ± 10.6	60.6 ± 10.1	56.6 ± 10.0	< 0.001	57.8 ± 10.0	61.1 ± 10.5	56.4 ± 10.8	< 0.001
Attended school until the age of 19 or higher (%)	20.2	22.3	17.6	< 0.001	10.9	11.9	9.4	0.14
Family history of colorectal cancer (%)^b^	2.1	2.4	2.3	0.62	2.7	2.7	2.4	0.81
Body mass index (kg/m^2^)^a^	23.0 ± 2.9	22.8 ± 3.1	22.4 ± 3.0	< 0.001	22.9 ± 3.4	23.3 ± 3.5	22.7 ± 3.5	0.004
Current alcohol drinkers (%)	68.8	74.2	77.1	< 0.001	21.5	43.6	43.8	< 0.001
Daily walking time 30+ min. (%)	68.1	66.8	69.6	< 0.001	71.2	66.1	69.4	0.016
Sedentary work (%)	32.8	34.3	32.5	0.063	35.7	31.3	33.9	0.086
Consumption of green leafy vegetables, every day (%)	31.6	31.1	26.8	< 0.001	34.4	34.5	27.4	< 0.001
Consumption of beef 3+ times/week (%)	8.8	8.5	9.3	0.16	10.9	12.2	10.6	0.61

^a^ Values are means ± standard deviation

^b^ Family history in parents and/or siblings.

We found a non-significant increase in age-adjusted risk of colon cancer in male ex- or current smokers compared with non-smokers: the HR values (HR1 in Table 2) were 1.23 (95% CI: 0.83-1.82) for ex-smokers and 1.23 (95% CI: 0.86-1.77) for current smokers. Further adjustment for potential confounding factors decreased the HR for male ex-smokers to 1.07 (HR2, 95% CI: 0.72-1.59) but did not alter the result for current smokers (HR2 1.23, 95% CI: 0.85-1.78). Almost no increase in colon cancer risk was detected for female smokers: the multivariate HR values (HR2) were 1.07 (95% CI: 0.39-2.92) for former smokers and 1.06 (95% CI: 0.55-2.02) for current smokers.

**Table 2.** Hazard ratios (HR) for cancers of the colon and rectum according to smoking status at baseline by sex.

Sex	Smoking status	Colon						Rectum					
	
		Person- years	No. of cases	HR1^a^	95% CI^b^	HR2^c^	95% CI^b^	Person- years	No. of cases	HR1^a^	95% CP	HR2^c^	95% CI^b^
Men	Never	40,436	39	1.00		1.00		40,436	34	1.00		1.00	
	Ex-smokers	50,079	67	1.23	0.83 - 1.82	1.07	0.72 - 1.59	50,079	44	0.94	0.60 - 1.47	0.88	0.56 - 1.39
	Current smokers	101,320	113	1.23	0.86 - 1.77	1.23	0.85 - 1.78	101,320	69	0.86	0.57 - 1.30	0.83	0.55 - 1.26
Women	Never	247,048	175	1.00		1.00		247,048	55	1.00		1.00	
	Ex-smokers	3,871	4	1.20	0.44 - 3.23	1.07	0.39 - 2.92	3,871	1	1.03	0.14 - 7.46	1.05	0.14 - 7.69
	Current smokers	13,443	10	1.13	0.60 - 2.15	1.06	0.55 - 2.02	13,443	1	0.36	0.05 - 2.57	0.36	0.05 - 2.65

^a^ HR1; adjusted for age.

^b^ CI: confidence interval.

^c^ HR2: adjusted for age, area (Hokkaido and Tohoku, Kanto, Chubu, Kinki, Chugoku, or Kyushu), education (attended school until the age of <16,16-18,or 19+), family history of colorectal cancer in parents or siblings (yes or no), body mass index (<20.0, 20.0-24.9, or 25.0+ kg/m^2^ for men, and <20.0, 20.0-24.9, 25.0-29.9, or 30.0+ kg/m^2^ for women), alcohol drinking (never drinkers, ex-drinkers, light current drinkers [<2.0 Japanese drinks/day], or heavy current drinkers [2.0+ Japanese drinks/day] for men, and never drinkers, ex-drinkers, or current drinkers for women), walking time (≤30 or 30+ minutes/day), sedentary work (yes or no), and consumption of green leafy vegetables (≤ 2 times/week, 3-4 times/week, or every day) and beef (almost never,1-2 times/month,1-2 times/week, or 3+ times/week).

For rectal cancer, male ex- or current smokers were not at an enhanced risk: the HRs adjusted for potential confounders (HR2) were 0.88 (95% CI: 0.56-1.39) and 0.83 (95% CI: 0.55-1.26) in ex- and current smokers, respectively. Only two female former or current smokers developed cancer of the rectum, which precluded us from precisely estimating the risk. We repeated the analyses in Table 2 after excluding the first two years of follow-up from the risk period but the findings remained essentially unchanged (data not shown).

The risk for male colon cancer was further assessed according to several characteristics of smoking habits in Table 3. We failed to observe an evident dose-response relationship between colon cancer risk and the number of cigarettes smoked per day, age at starting smoking, years of smoking, or cumulative amount of smoking. Higher HRs were associated with moderate exposure rather than heavy smoking. For example, men with 20-39 pack-years of smoking at baseline demonstrated an elevated risk with marginal significance (HR2 1.43, 95% CI: 0.98-2.10) but the HR was lower in men with more cumulative consumption. The fully-adjusted HR (HR2) for 40+ years of smoking was 1.07 (95% CI: 0.71-1.61) and was rather smaller than the ratio for 20-39 years of smoking. In addition, we did not find a decreasing trend in risk with increasing years after smoking cessation.

**Table 3.** Hazard ratios (HR) for colon cancer according to characteristics of smoking habits at baseline in men.

Smoking habits	Person- years	No. of cases	HR1^a^	95% CI^b^	HR2^c^	95% CI^b^
No. of cigarettes smoked per day						
Never	40,436	39	1.00		1.00	
0-19	47,854	59	1.14	0.76 - 1.70	1.05	0.70 - 1.58
20-39	83,369	102	1.34	0.93 - 1.94	1.30	0.89 - 1.89
40+	14,317	9	0.76	0.37 - 1.58	0.69	0.33 - 1.43
				Trend p = 0.43		Trend p = 0.56
Age at starting smoking (years)						
Never	40,436	39	1.00		1.00	
26+	12,749	18	1.15	0.66 - 2.02	1.10	0.62 - 1.93
23-25	19,771	34	1.66	1.05 - 2.63	1.54	0.97 - 2.44
20-22	84,195	97	1.21	0.84 - 1.76	1.13	0.78 - 1.64
<20	28,513	24	1.01	0.61 - 1.69	1.04	0.62 - 1.74
				Trend p = 0.64		Trend p = 0.76
Years of smoking						
Never	40,436	39	1.00		1.00	
0-19	16,417	13	1.14	0.61 - 2.14	0.99	0.53 - 1.87
20-39	86,485	92	1.42	0.97 - 2.09	1.31	0.89 - 1.92
40+	41,078	67	1.08	0.72 - 1.62	1.07	0.71 - 1.61
				Trend p = 0.47		Trend p = 0.52
Cumulative amount of smoking (pack-years)						
Never	40,436	39	1.00		1.00	
0-19	30,048	26	1.04	0.63 - 1.71	0.92	0.56 - 1.52
20-39	66,489	89	1.52	1.04 - 2.21	1.43	0.98 - 2.10
40-59	32,239	41	1.11	0.72 - 1.73	1.11	0.71 - 1.73
60+	11,908	10	0.72	0.36 - 1.44	0.68	0.34 - 1.37
				Trend p = 0.87		Trend p = 0.90
Years since smoking cessation						
Never	40,436	39	1.00		1.00	
Current smokers	101,320	113	1.23	0.86 - 1.78	1.23	0.85 - 1.78
Ex-smokers (years)						
0-9	24,916	31	1.21	0.76 - 1.94	1.09	0.68 - 1.75
10-19	14,302	23	1.54	0.92 - 2.57	1.29	0.77 - 2.17
20+	9,618	12	0.95	0.50 - 1.83	0.79	0.41 - 1.52
				Trend p = 0.86^d^		Trend p = 0.29^d^

^a^ HR1: adjusted for age.

^b^ CI: confidence interval.

^c^ HR2: adjusted for age, area (Hokkaido and Tohoku, Kanto, Chubu, Kinki, Chugoku, or Kyushu), education (attended school until the age of <16, 16-18, or 19+), family history of colorectal cancer in parents or siblings (yes or no), body mass index (<20.0, 20.0-24.9, or 25.0+ kg/m^2^ for men, and <20.0, 20.0-24.9, 25.0-29.9, or 30.0+ kg/m^2^ for women), alcohol drinking (never drinkers, ex-drinkers, light current drinkers [<2.0 Japanese drinks/day], or heavy current drinkers [2.0+ Japanese drinks/day] for men, and never drinkers, ex-drinkers, or current drinkers for women), walking time (≤30 or 30+ minutes/day), sedentary work (yes or no), and consumption of green leafy vegetables (≤2 times/week, 3-4 times/week, or every day) and beef (almost never, 1-2 times/month, 1-2 times/week, or 3+ times/week).

^d^ Trend for ex- and current smokers.

No significant association was found between the risk of rectal cancer and various characteristics of the smoking habit at baseline (Table 4). There appeared somewhat decreasing trends in risk with the increasing number of cigarettes smoked per day or increasing cumulative measure of smoking, but the trends were far from significant. The analysis dividing cumulative amount of smoking at age of 30 years or at 20 years before baseline in men revealed no clear trends in risk of colon or rectal cancer according to cigarette pack-years in either of the two periods, although men who had smoked 10 pack-years or more after age 30 years showed a slightly higher risk (Table 5).

**Table 4.** Hazard ratios (HR) for rectal cancer according to characteristics of smoking habits at baseline in men.

Smoking habits	Person- years	No. of cases	HR1^a^	95% CI^b^	HR2^c^	95% CI^b^
No. of cigarettes smoked per day						
Never	40,436	34	1.00		1.00	
0-19	47,854	44	0.98	0.63 - 1.54	0.95	0.60 - 1.50
20-39	83,369	55	0.83	0.54 - 1.27	0.79	0.51 - 1.22
40+	14,317	9	0.87	0.42 - 1.81	0.80	0.38 - 1.69
				Trend p = 0.37		Trend p = 0.26
Age at starting smoking (years)						
Never	40,436	34	1.00		1.00	
26+	12,749	10	0.74	0.36 - 1.50	0.73	0.36 - 1.49
23-25	19,771	16	0.90	0.50 - 1.63	0.84	0.46 - 1.53
20-22	84,195	56	0.81	0.53 - 1.23	0.77	0.50 - 1.18
<20	28,513	25	1.21	0.72 - 2.04	1.18	0.69 - 1.99
				Trend p = 0.95		Trend p = 0.91
Years of smoking						
Never	40,436	34	1.00		1.00	
0-19	16,417	6	0.59	0.25 - 1.42	0.58	0.24 - 1.39
20-39	86,485	61	1.06	0.69 - 1.63	1.01	0.65 - 1.56
40+	41,078	39	0.74	0.46 - 1.19	0.72	0.45 - 1.16
				Trend p = 0.45		Trend p = 0.35
Cumulative amount of smoking (pack-years)						
Never	40,436	34	1.00		1.00	
0-19	30,048	22	1.00	0.58 - 1.71	0.96	0.56 - 1.66
20-39	66,489	48	0.93	0.60 - 1.44	0.89	0.57 - 1.40
40-59	32,239	24	0.76	0.45 - 1.29	0.72	0.42 - 1.22
60+	11,908	10	0.84	0.42 - 1.71	0.78	0.38 - 1.59
				Trend p = 0.33		Trend p = 0.23
Years since smoking cessation						
Never	40,436	34	1.00		1.00	
Current smokers	101,320	69	0.86	0.57 - 1.30	0.83	0.55 - 1.26
Ex-smokers (years)						
0-9	24,916	16	0.72	0.40 - 1.31	0.68	0.37 - 1.24
10-19	14,302	20	1.55	0.89 - 2.70	1.47	0.84 - 2.57
20+	9,618	6	0.56	0.23 - 1.33	0.53	0.22 - 1.28
				Trend p = 0.71^d^		Trend p = 0.80^d^

^a^ HR1: adjusted for age.

^b^ CI: confidence interval.

^c^ HR2: adjusted for age, area (Hokkaido and Tohoku, Kanto, Chubu, Kinki, Chugoku, or Kyushu), education (attended school until the age of <16, 16-18, or 19+), family history of colorectal cancer in parents or siblings (yes or no), body mass index (<20.0, 20.0-24.9, or 25.0+ kg/m^2^ for men, and <20.0, 20.0-24.9, 25.0-29.9, or 30.0+ kg/m^2^ for women), alcohol drinking (never drinkers, ex-drinkers, light current drinkers [<2.0 Japanese drinks/day], or heavy current drinkers [2.0+ Japanese drinks/day] for men, and never drinkers, ex-drinkers, or current drinkers for women), walking time (≤30 or 30+ minutes/day), sedentary work (yes or no), and consumption of green leafy vegetables (≤2 times/week, 3-4 times/week, or every day) and beef (almost never, 1-2 times/month, 1-2 times/week, or 3+ times/week).

^d^ Trend for ex- and current smokers.

**Table 5.** Hazard ratios (HR) for cancers of the colon and rectum according to cumulative amount of smoking during different age and time periods in men.

Cumulative amount of smoking (pack-years)	Colon						Rectum					
	
	Person- years	No. of cases	HR1^a^	95% CI^b^	HR2^c^	95% CI^b^	Person- years	No. of cases	HR1^a^	95% CI^b^	HR2^c^	95% CI^b^
Up to age 30 years												
None	50,109	53	1.00		1.00		50,109	42	1.00		1.00	
0.0-9.9	60,371	76	1.16	0.81 - 1.64	1.09	0.76 - 1.55	60,371	50	0.96	0.64 - 1.45	0.94	0.62 - 1.42
10.0-19.9	60,235	70	1.32	0.92 - 1.89	1.28	0.89 - 1.85	60,235	40	0.93	0.60 - 1.44	0.88	0.56 - 1.36
20.0+	11,108	6	0.68	0.29 - 1.59	0.63	0.27 - 1.48	11,108	6	0.83	0.35 - 1.96	0.78	0.33 - 1.85
				Trend p = 0.49		Trend p = 0.62				Trend p = 0.64		Trend p = 0.47

After age 30 years												
None	44,809	39	1.00		1.00		44,809	36	1.00		1.00	
0.0-9.9	16,079	13	1.17	0.62 - 2.20	1.05	0.56 - 1.97	16,079	12	1.16	0.60 - 2.23	1.13	0.58 - 2.19
10.0-19.9	40,523	41	1.46	0.94 - 2.28	1.35	0.87 - 2.11	40,523	27	1.03	0.62 - 1.70	0.99	0.59 - 1.64
20.0+	80,410	112	1.37	0.95 - 1.97	1.35	0.93 - 1.95	80,410	63	0.84	0.56 - 1.27	0.81	0.53 - 1.22
				Trend p = 0.089		Trend p = 0.093				Trend p = 0.35		Trend p = 0.26
Up to 20 years before baseline												
None	49,563	43	1.00		1.00		49,563	36	1.00		1.00	
0.0-9.9	42,749	29	1.06	0.65 - 1.71	0.99	0.61 - 1.61	42,749	22	0.93	0.54 - 1.60	0.90	0.52 - 1.56
10.0-19.9	44,712	69	1.57	1.07 - 2.30	1.50	1.02 - 2.21	44,712	37	1.01	0.64 - 1.60	0.98	0.62 - 1.57
20.0+	44,798	64	1.11	0.74 - 1.64	1.08	0.72 - 1.61	44,798	43	0.92	0.58 - 1.45	0.87	0.55 - 1.38
				Trend p = 0.36		Trend p = 0.44				Trend p = 0.77		Trend p = 0.62
Within 20 years of baseline												
None	48,983	49	1.00		1.00		48,983	39	1.00		1.00	
0.0-9.9	18,026	30	1.62	1.03 - 2.56	1.47	0.93 - 2.32	18,026	27	1.85	1.13 - 3.02	1.78	1.08 - 2.91
10.0-19.9	44,606	55	1.23	0.83 - 1.80	1.21	0.82 - 1.79	44,606	29	0.81	0.50 - 1.31	0.79	0.49 - 1.29
20.0+	70,207	71	1.22	0.85 - 1.77	1.28	0.88 - 1.86	70,207	43	0.90	0.58 - 1.40	0.86	0.55 - 1.35
				Trend p = 0.44		Trend p = 0.29				Trend p = 0.25		Trend p = 0.19

^a^ HR1: adjusted for age.

^b^ CI: confidence interval.

^c^ HR2: adjusted for age, area (Hokkaido and Tohoku, Kanto, Chubu, Kinki, Chugoku, or Kyushu), education (attended school until the age of <16, 16-18, or 19+), family history of colorectal cancer in parents or siblings (yes or no), body mass index (<20.0, 20.0-24.9, or 25.0+ kg/m^2^ for men, and <20.0, 20.0-24.9, 25.0-29.9, or 30.0+ kg/m^2^ for women), alcohol drinking (never drinkers, ex-drinkers, light current drinkers [<2.0 Japanese drinks/day], or heavy current drinkers [2.0+ Japanese drinks/day] for men, and never drinkers, ex-drinkers, or current drinkers for women), walking time (≤30 or 30+ minutes/day), sedentary work (yes or no), and consumption of green leafy vegetables (≤2 times/week, 3-4 times/week, or every day) and beef (almost never, 1-2 times/month, 1-2 times/week, or 3+ times/week).

## DISCUSSION

In the present large prospective study of Japanese men and women, we observed no appreciable increase in the risk of colon cancer in current smokers for both sexes. We found no clear dose-response relationship. Furthermore, male smokers showed no excess risk of rectal cancer.

Our findings imply that smoking is not a major causative factor of colorectal cancer, although the possibility of a weak association cannot be excluded. Most studies conducted in Western countries other than North America,^5^^,^^14^^–^^16^ though not all,^17^ are in general agreement with our findings. For example, Nyrén et al.^5^ performed a 20-year follow-up study of 135,000 male construction workers in Sweden and found no increased risk of colon cancer even in long-term heavy smokers. They observed a non-significant, 20% increase in rectal cancer risk in smokers, but detected no dose-response relationship. D’Avanzo et al.^15^ conducted a case-control study involving 1,584 cases of colorectal cancer and 2,879 controls, and indicated that smoking was not a strong risk factor for the cancer even after a long induction period.

The sex (male-to-female) ratio of incidence for colon cancer was 1.60 in the present study. If the male higher proportion of ever smokers in Japan explains the sex difference in incidence, the HR for ever smokers may be estimated to be 1.88 from the following equation:

Sex ratio = {(l-p_m_)+p_m_ · HR}/{(l-p_f_)+p_f_ · HR},

where p_m_ and p_f_ were proportions of ever smokers in males (79.4%) and females (6.9%) in the cohort, respectively.

The HRs for smokers in the present study ranged from 1.13 (female current smokers) to 1.23 (male ex- or current smokers) for age-adjusted ratios and from 1.06 (female current smokers) to 1.23 (male current smokers) for multivariate-adjusted ratios (Table 2). These are considerably lower than that estimated from the sex ratio of incidence and the percentages of ever smokers. This gap might suggest the irrelevance of smoking to colon cancer risk.

A relatively small number of studies previously examined the role of smoking in development of colorectal cancer among non-Western populations and the results were inconsistent. Akiba and Hirayama^18^ reported an increase in risk for male rectal cancer but not for male colon cancer or female colon/rectal cancer in a large-scale cohort study in Japan. The relative risk for daily smokers was 1.4 (95% CI: 1.0-1.9) for cancers of the rectum in men. Shimizu et al., in another prospective study,^19^ reported a significantly increased risk of rectal cancer in men who had smoked more than 20 pack-years of cigarettes. A hospital-based case-control study, also in Japan,^20^ found that habitual smoking was associated with an increased risk of rectal cancer in both sexes but not that for colon cancer. In another case-control study in the same country,^21^ cumulative amount of cigarette smoking until 20 years before the diagnosis was significantly associated with the risk of colorectal cancer. On the other hand, Hoshiyama and his colleagues^22^ reported a decreased risk of colon cancer in relation to cigarette smoking. Case-control studies in Seoul^23^ and Shanghai^24^ furthermore concluded that smoking in general was not a risk factor for the cancers of colon and rectum. Our results are in line with these inconsistent findings in Asian countries.

Smoking might primarily affect early stages in the development of colorectal cancer.^4^^,^^21^^,^^25^ The relatively high age at starting smoking,^26^ therefore, might provide an explanation for the inconsistent results in Japan. We performed analyses focusing on smoking in the distant past, but could not detect specific effects. Unfortunately, cigarette smoking has now become more prevalent in Japanese teenagers,^27^ which might increase the risk in the future.

An increased risk of colorectal cancer has been found among long-term smokers in several cohort studies in the United States,^3^ including the Health Professionals Follow-up Study,^4^ a follow-up study of US veterans,^25^ the Physicians’ Health Study I,^28^ and the Cancer Prevention Study II.^29^ The reasons for the discrepancies between results of these investigations and the majority of reports in other countries^5^^,^^14^^–^^16^^,^^22^^–^^24^ remain unclear. One possible explanation, however, is that American cigarettes are more toxic and contain more carcinogens in their mainstream smoke.^26^ Another reason would be that more health professional smokers^4^^,^^28^ had stopped smoking than in the general population and those who did not quit might be more addicted smokers with a tendency to inhale more deeply and take more puffs per cigarette.^30^

The strengths of the present study are its prospective design and the large sample size. We assessed smoking habits and other variables before the diagnosis of colorectal cancer; thus any errors of recall should have been non-differential between cases and non-cases. A considerable number of cases of colon and rectal cancers were identified in male former or current smokers, which enabled us to assess the risk in relation to detailed smoking habits.

Some methodological limitations, however, do need consideration. First, information on tobacco use was not updated after the baseline survey. If smokers at baseline quit smoking during the follow-up period, the association of current smoking with colorectal cancer risk may be weakened. For part of the present subjects (8,843 men and 12,487 women), we undertook a follow-up questionnaire survey on lifestyles after five years from baseline (mean ± standard deviation: 4.9 ± 0.6 years) and elucidated their smoking status. Of current smokers at baseline, 16.0% of men and 11.1% of women reported themselves as former smokers at the second survey. Such smoking cessation would have resulted in a somewhat attenuated risk for current smokers. Although cohort studies on smoking and the risk of colorectal cancer usually assess smoking habits of the participants only at baseline, studies with repeated measurements^4^ may provide more meaningful information.

Second, although the study period was reasonably long, extended follow-up may reveal increase in colorectal cancer risk among long-term smokers. Our cohort, however, included the sufficient number of men with 40+ years of smoking already at baseline, in whom some previous studies found an increased risk.^4^^,^^29^

The third limitation may be that we cannot exclude the possibility of residual confounding although we adjusted for selected risk or protective factors. No information was available on some factors such as aspirin use.^31^ Our questionnaire could not be used to estimate energy expenditure.^12^

Finally, the confidence interval for HR was relatively large. For colon cancer, the upper limit of 95% CI for multivariate-adjusted HR for current smokers versus never smokers was 1.78 in men and 2.02 in women (HR2 in Table 2). We therefore cannot exclude a possible weak association (i.e., an association with a relative risk less than two) between smoking and colon cancer reported in some studies.^19^^,^^25^

In conclusion, our cohort study in Japan, in line with many previous investigations,^5^^,^^14^^–^^16^^,^^22^^–^^24^ indicates that cigarette smoking is not a strong risk factor for colorectal cancer. As suggested for male colon cancer here, however, a weak association remains open to discussion. We also need to identify the reasons for the discrepancies in comparison with several studies in the United States,^4^^,^^25^^,^^28^^,^^29^ which showed an increased risk of colorectal cancer, particularly in long-term smokers.

## APPENDIX

The present members of the JACC Study and their affiliations are as follows: Dr. Akiko Tamakoshi (present chairman of the study group), Nagoya University Graduate School of Medicine; Dr. Mitsuru Mori, Sapporo Medical University School of Medicine; Dr. Yutaka Motohashi, Akita University School of Medicine; Dr. Ichiro Tsuji, Tohoku University Graduate School of Medicine; Dr. Yosikazu Nakamura, Jichi Medical School; Dr. Hiroyasu Iso, Institute of Community Medicine, University of Tsukuba; Dr. Haruo Mikami, Chiba Cancer Center; Dr. Yutaka Inaba, Juntendo University School of Medicine; Dr. Yoshiharu Hoshiyama, Showa University School of Medicine; Dr. Hiroshi Suzuki, Niigata University Graduate School of Medical and Dental Sciences; Dr. Hiroyuki Shimizu, Gifu University School of Medicine; Dr. Hideaki Toyoshima, Nagoya University Graduate School of Medicine; Dr. Shinkan Tokudome, Nagoya City University Graduate School of Medicine; Dr. Yoshinori Ito, Fujita Health University School of Health Sciences; Dr. Shuji Hashimoto, Fujita Health University School of Medicine; Dr. Shogo Kikuchi, Aichi Medical University School of Medicine; Dr. Akio Koizumi, Graduate School of Medicine and Faculty of Medicine, Kyoto University; Dr. Takashi Kawamura, Kyoto University Center for Student Health; Dr. Yoshiyuki Watanabe and Dr. Tsuneharu Miki, Kyoto Prefectural University of Medicine Graduate School of Medical Science; Dr. Chigusa Date, Faculty of Human Environmental Sciences, Mukogawa Women’s University ; Dr. Kiyomi Sakata, Wakayama Medical University; Dr. Takayuki Nose, Tottori University Faculty of Medicine; Dr. Norihiko Hayakawa, Research Institute for Radiation Biology and Medicine, Hiroshima University; Dr. Takesumi Yoshimura, Institute of Industrial Ecological Sciences, University of Occupational and Environmental Health, Japan; Dr. Katsuhiro Fukuda, Kurume University School of Medicine; Dr. Naoyuki Okamoto, Kanagawa Cancer Center; Dr. Hideo Shio, Moriyama Municipal Hospital; Dr. Yoshiyuki Ohno (former chairman of the study group), Asahi Rosai Hospital; Dr. Tomoyuki Kitagawa, Cancer Institute of the Japanese Foundation for Cancer Research; Dr. Toshio Kuroki, Gifu University; and Dr. Kazuo Tajima, Aichi Cancer Center Research Institute.

The past investigators of the study group were listed in reference 7 except for the following seven members (affiliations are those at the time they participated in the study): Dr. Takashi Shimamoto, Institute of Community Medicine, University of Tsukuba; Dr. Heizo Tanaka, Medical Research Institute, Tokyo Medical and Dental University; Dr. Shigeru Hisamichi, Tohoku University Graduate School of Medicine; Dr. Masahiro Nakao, Kyoto Prefectural University of Medicine; Dr. Takaichiro Suzuki, Research Institute, Osaka Medical Center for Cancer and Cardiovascular Diseases; Dr. Tsutomu Hashimoto, Wakayama Medical University; and Dr. Teruo Ishibashi, Asama General Hospital.
